# Supplementary material for: Determining the patency of biliary tracts in dogs with gallbladder mucocele using near-infrared cholangiography with indocyanine green
Source: PLoS One. 2024 May 22;19(5):e0300395. doi: 10.1371/journal.pone.0300395 (PMC11111041; doi:10.1371/journal.pone.0300395)
Supplement: S1 Fig — (PDF) [file pone.0300395.s001.pdf]

GB	Normal	GBM	
		Mobile	Immobile
Patient number	Fluoresence Intensity		
1	16350	4662	805
2	15264	4028	791
3	14947	3704	692
4	16300	3861	559
5	14582	5098	762
6	16222	5613	924
7	15478		656
8	16222		692
9	15848		919
10	14478		540
11			753
Average	15569.1	4494.33333	735.727273
Standard Deviation	687.57697	693.679241	119.696655

CBD	Normal	GBM	
		Mobile	Immobile
Patient number	Fluoresencre Intensity		
1	16248	15673	15326
2	15499	15423	15623
3	15761	15224	14463
4	16020	13964	14632
5	15092	15293	16342
6	16383	15673	14743
7	15542		15634
8	16234		15923
9	15429		14462
10	16092		15934
11			15523
Average	15830	15208.3333	15327.7273
Standard Deviation	406.350095	582.192885	624.715803
