# Supplementary material for: Determining the patency of biliary tracts in dogs with gallbladder mucocele using near-infrared cholangiography with indocyanine green
Source: PLoS One. 2024 May 22;19(5):e0300395. doi: 10.1371/journal.pone.0300395 (PMC11111041; doi:10.1371/journal.pone.0300395)
Supplement: Attachment — Submitted filename: [GB]Response to Reviewers_Review2.docx [file pone.0300395.s002.docx]

**[Reviewer #2]**

*** Major revisions**

1. **The full name of CBD should be supplemented in the abstract.**

**Response:** We thank the reviewer for this suggestion, and we introduced the necessary changes (page 2, line 38 in the revised manuscript).

2. **Tolerance of humans and animals to the same drug is very different. Generally speaking, the drug tolerance of animals is larger than people, which means animals need higher drug doses than humans. So, has the author adjusted the drug dose (0.25mg/kg was used in the study)?**

**Response:** We appreciate your valuable suggestion. As stated in the discussion (lines 355-360), we determined the final dosage based on various references from human medicine. Specifically, one study referenced in our discussion (Reference 26) utilized dosages ranging from 0.05 to 0.25 mg/kg for cholangiography in healthy dogs. Upon comparing our results with this study, the dosage of 0.25 mg/kg appeared suitable for evaluating patency in both studies (Page 16, lines 360-366 in the revised manuscript). However, considering pathological conditions and other factors, further investigation is warranted to determine the optimal dose for dogs.

3. **Where is the scale bar of Figure 3?**

**Response:** We appreciate your valuable suggestion. Unfortunately, the imaging device used in our study does not provide a scale reference for real-time visualization of organ sizes. While we did measure the size of the excised gallbladders post-surgery, comparing them to the intraoperative images may introduce inaccuracies due to perspective distortion. Therefore, we could not include a scale bar in Figure 3 to avoid potential inaccuracies in size estimation.

4. **Whether the authors measure the residual amount of ICG in other organs, such as the kidney, heart, spleen, and lung?**

**Response:** Thank you for the reviewer’s comment. In this study, our primary objective was to confirm the patency of the biliary tract and assess biliary tree visualization using cholangiography rather than measuring the residual amount of ICG in other organs. As such, our focus was directed towards the specific aims of this investigation. However, we acknowledge the importance of exploring the residual amount of ICG in other organs as a potential avenue for further research. Future studies could indeed delve into this aspect to provide a more comprehensive understanding of the pharmacokinetics and biodistribution of ICG in various tissues.

5. **Is there any difference in the results between different dog types, especially between large and small dogs?**

**Response:** We appreciate your inquiry. Upon reviewing the results of our study, we found minimal differences between different dog types, as indicated in Table 1. It was challenging to draw conclusive comparisons based solely on breed differences. Further investigation into potential variations between dog types, particularly between large and small breeds, may be warranted to provide a more comprehensive understanding of the implications of breed-specific factors on the outcomes of NIR cholangiography.

6. **This research only studied whether IOC can be applied in canine cholangiography, there are limited experiments and data. I suggest more investigation into the dose of ICG administration and the detection time after ICG administration because these supplements in the experiment improve the scientific significance and reference value of this article.**

**Response:** We appreciate your insightful suggestions. We are fully aware of the importance of further investigation into the dose of ICG administration and the detection time after ICG administration. These aspects are currently under consideration, and we are planning to incorporate them into our ongoing research efforts. Your suggestions will undoubtedly contribute to the refinement and advancement of our study, and we are committed to addressing them in our future work.

7. **Has the author tested the feasibility of this method on other animals?**

**Response:** We appreciate your valuable comment. In this study, we did not conduct feasibility testing on other animals. Instead, we relied on references from previous studies involving humans, or dogs to establish the method's safety and feasibility (see references below). Considering the established safety based on these references, additional experiments on other animal species were not pursued in this study. Nonetheless, we closely monitored the canine patients both immediately after injection and post-surgery, and no adverse reactions related to ICG were observed during this study.

References:

1. Zarrinpar A, Dutson EP, Mobley C, Busuttil RW, Lewis CE, Tillou A, et al. Intraoperative Laparoscopic Near-Infrared Fluorescence Cholangiography to Facilitate Anatomical Identification: When to Give Indocyanine Green and How Much. Surg Innov. 2016;23(4): 360-5.
2. HOPE-ROSS, Monique, et al. Adverse reactions due to indocyanine green. Ophthalmology, 1994;101(3): 529-533.
3. Larose PC, Brisson BA, Sanchez A, Monteith G, Singh A, Zhang M. Near-infrared fluorescence cholangiography in dogs: A pilot study. Vet Surg. 2023.
4. GENÉ ŠKRABEC, Clara, et al. Fluorescent cholangiography with direct injection of indocyanine green (ICG) into the gallbladder: a safety method to outline biliary anatomy. *Langenbeck's Archives of Surgery*, 2020;405: 827-832.
8. **There is a clerical error on Page 5 line 121 “0.25/mL”.**

**Response:** We thank the reviewer for pointing this out, and we apologize for this error. We revised this sentence (page 5, line 121 in the revised manuscript).

9. **The author should pay attention to the space between numbers, symbols, and letters in the figure legends. For example, “n=6”, and “p=0.002” in page 7.**

**Response:** We appreciate the useful comments and criticisms provided by the reviewer, and, following a long and careful discussion, we agree with all the reviewer’s suggestions, and have introduced the appropriate changes in the remainder of the manuscript.
